# Deterministic inference for stochastic systems using multiple shooting and a linear noise approximation for the transition probabilities

**DOI:** 10.1049/iet-syb.2014.0020

**Published:** 2015-10-01

**Authors:** Christoph Zimmer, Sven Sahle

**Affiliations:** ^1^ BIOMS University of Heidelberg Im Neuenheimer Feld 368 69120 Heidelberg Germany; ^2^ BioQuant University of Heidelberg Im Neuenheimer Feld 267 69120 Heidelberg Germany

**Keywords:** stochastic systems, parameter estimation, probability, least squares approximations, deterministic inference, stochastic systems, multiple shooting, linear noise approximation, transition probabilities, systems biology, parameter estimation methods, likelihood function, normal distributions, intrinsic stochasticity effects, reversible jump techniques, approximative sampling, conventional least‐squares parameter estimation methods

## Abstract

Estimating model parameters from experimental data is a crucial technique for working with computational models in systems biology. Since stochastic models are increasingly important, parameter estimation methods for stochastic modelling are also of increasing interest. This study presents an extension to the ‘multiple shooting for stochastic systems (MSS)’ method for parameter estimation. The transition probabilities of the likelihood function are approximated with normal distributions. Means and variances are calculated with a linear noise approximation on the interval between succeeding measurements. The fact that the system is only approximated on intervals which are short in comparison with the total observation horizon allows to deal with effects of the intrinsic stochasticity. The study presents scenarios in which the extension is essential for successfully estimating the parameters and scenarios in which the extension is of modest benefit. Furthermore, it compares the estimation results with reversible jump techniques showing that the approximation does not lead to a loss of accuracy. Since the method is not based on stochastic simulations or approximative sampling of distributions, its computational speed is comparable with conventional least‐squares parameter estimation methods.

## 1 Introduction

Parameter estimation is very important for the analysis of models in systems biology. Computational modelling is a central approach in systems biology for studying increasingly complex biochemical systems. Progress in experimental techniques, for example, the possibility to measure small numbers of molecules in single cells [1], highlights the need for stochastic modelling approaches. Simulation methods for stochastic processes are being developed for decades since [2] and nowadays exist with a lot of variants [3]. The development of parameter estimation methods for stochastic models, however, has started only recently. This paper extends a method of [4] with a more accurate approximation for the variances of the transition probabilities.

Parameter estimation approaches for time series data exist using stochastic simulations. Owing to the Markov property of the time series, the likelihood function factorises into the product of transition probabilities. These transition probabilities are generally unknown in stochastic modelling. They can be estimated using stochastic simulations. This can be done with density estimation methods [5, 6]. Another approach is the use of a reversible jump algorithm [7, 8]. As results of these two papers will be used for comparison, their ideas will be explained in more detail. Reversible jump algorithms start with a path connecting the previous and the succeeding state that contains the minimum number of reactions necessary to move from state to state. For this specific path it is possible to calculate the probability. Then reactions are added and subtracted from the path to obtain new paths and their probabilities. The transition probability is the sum over all possible paths. The space of paths is explored in a Monte Carlo way. The parameter estimation can then be performed with Bayesian methods as in [7]. The same idea of exploring the space of possible paths can also be used to calculate derivatives of the transition probabilities with respect to the parameter. This can be combined with a gradient descent for the optimisation as in [8]. Using a surrogate probabilistic model as an approximation is faster from a computational point of view [9]. Komorowski *et al.* [10] approximates the system with a multivariate normal distribution, in which the mean is described by the rate equations and the covariance matrix contains all inter‐temporal covariances. Another approximation is suggested in form of an approximate maximum‐likelihood method [11], where also a singular value decomposition likelihood method is described. A use of approximate Bayesian methods is suggested in [12].

A second class of methods focuses on a numerical solution of the chemical master equation (CME), which describes the probability for each state as a function of time. These systems are generally high dimensional. To address this problem, a state space truncation can be used [13] or moment‐closure methods, which are an approximation focusing on a finite number of moments of the probability distribution [14, 15]. Parameter estimation with the CME and an approximation for the likelihood function for small systems or a hybrid method for large systems is suggested in [16]. Deuflhard *et al.* [17] and Engblom [18] use an adaptive Galerkin method for the solution of the CME. If distribution information is available from measurement, a finite state projection [19] can be used to solve the CME. The common challenge is the fact that the solution of the CME, as well as simulation‐based methods, become very time‐consuming as the number of states in the state space increases.

Moment matching methods use repeated measurements to estimate moments of the distribution for the parameter estimation [20, 21]. Further approaches can be taken from stochastic epidemic models and are based on expectation maximisation algorithms or Martingale‐based approaches [22]. A different approach is suggested by Chattopadhyaya *et al.* [23], called ‘inverse Gillespie’, using certain geometrical properties of the population updated to infer the reaction structure and the propensities.

The problem for parameter estimation for stochastic models can be written in the following form: we assume that the experimental or simulated data records at each time point *t_i_*, *i* = 0, 1, …, *n*, the number of molecules of species *k*, $\nu_{i}^{\left(k\right)}$, hence $\nu_{i}^{\left(k\right)}\in N_{0}$. Each data set *ν* = (*ν*_0_, *ν*_1_, …, *ν_n_*) is stochastic. This means that even two simulated realisations of the system without measurement noise can be different, and even that the mean of stochastic simulations does not have to be close to the ordinary differential equation (ODE) solution. Furthermore, it is assumed that the system's behaviour depends on some unknown parameters *θ*, which are to be estimated. To this goal, the data *ν* are compared with some sort of stochastic or ODE simulations with respect to some objective function *F* which measures the quality of the fit. Parameter estimation means finding the optimal value $\hat{\theta }$ for the parameter.

A common choice for measuring the quality of a fit is the likelihood function. The likelihood function describes the probability for obtaining a data set *ν* given a parameter *θ*. A maximum‐likelihood estimator is the choice of *θ* that maximises the probability to obtain the data *ν*. For processes satisfying the Markov property, the likelihood function factorises into the product of the transition probabilities from a point *ν_i_*_−1_ at time *t_i_*_−1_ to a point *ν_i_* at time *t_i_*, *i* = 1, …, *n*.

The MSS approach that we recently developed [4] approximates this transition probability with a normal distribution of constant variance. It has been shown to work well on oscillatory systems even when the dynamics is qualitatively different from an ODE solution. However, certainly the approximation with constant variance is rough and can be improved. The new approach therefore approximates the transition probabilities with a normal distribution with a covariance calculated by a linear noise approximation (LNA) on the intervals between two succeeding time points of measurements. The conditions for the approximation on the shorter time intervals are much less strict than for the LNA applied over the complete time course.

Therefore the approach is comparable with [7, 8, 9] in that it uses a factorisation of the likelihood function, but is different from those in that it does not use simulations to calculate the transition probabilities.

The extended MSS method can tackle models with fully observed and partially observed data sets. The fact that it works without stochastic simulations and without solving a high‐dimensional CME means that it is possible to approach systems of a size as large as realistic models being approached with conventional deterministic methods. Since the evaluation of the objective function does not involve stochastic simulations, all methods from derivative‐based methods, to global optimisation techniques, to Bayesian methods can be applied for its numerical optimisation.

**Fig. 1:**
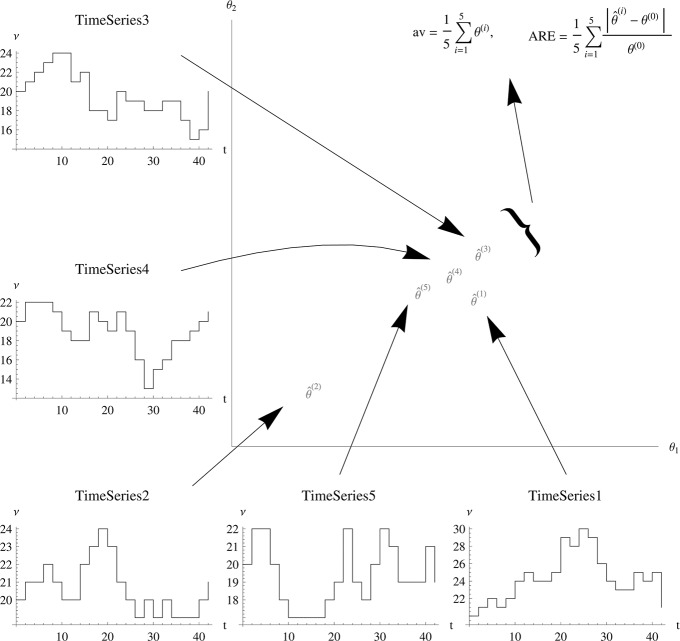
Outline of the simulation study Time courses are simulated (5 small graphics) and used as pseudo data for a parameter estimation (rep resented by the arrows). Each time series results in one estimator plotted in the coordinate system in the middle. As each time series is intrinsically stochastic, the estimators are random variables clustering around the true parameter value. To judge “how close” they are, two statistical quantities are displayed: their average av,. to see if the method is unbiased, and their average relative error ARE, to see how far they spread around the true value. Note that for a parameter estimation one time series is necessary. Only for the simulation study more than one time series is needed.

As the objective function of the MSS method is deterministic, the estimator for a single stochastic time course is unique. However, using the same model with the same parameters and initial conditions with a different stochastic time course realisation, the estimation will result in a different value because of the intrinsic stochasticity of the time courses. To evaluate the performance of the objective function, it is therefore necessary to estimate more than one stochastic realisation of the same model with the same parameters and initial conditions. This paper uses 100 different realisations for each model. A statistic over the 100 estimations can then be used to assess the quality of the objective function (Fig. 1). It has to be underlined that the 100 realisations are only used to test the quality of the objective function. For an estimation one realisation is sufficient.

This paper is structured as follows:

The method section will introduce stochastic modelling, suggest and discuss the approximation, and present the resulting objective function.

The results section will show the performance of the extended MSS method on stochastic models of an immigration‐death model, a prokaryotic auto‐regulatory network and a Lotka–Volterra model. For the different scenarios, we will discuss the performance of the extended MSS method with respect to a state‐of‐the‐art alternative – namely reversible jump techniques – and the old MSS approach.

## 2 Method

### 2.1 Stochastic modelling of biochemical reactions

This section will introduce stochastic modelling and explain in which situations it is important. Let *X* = (*X*_1_, …, *X_D_*) denote the *D* reactants in a system with *r* reactions in which *q_ij_* denotes the number of educts or reactant molecules of species *X_i_* for reaction *j* and *u_ij_* is the number of product molecules of species *X_i_* for reaction *j*. Hence, the systems read as

$$\begin{array}{rl} & q_{1j}X_{1}+q_{2j}X_{2}+\cdots +q_{Dj}X_{D}\\ & \;\to u_{1j}X_{1}+u_{2j}X_{2}+\cdots +u_{Dj}X_{D},\;\text{for}\;\;j=1,\;\ldots ,\;r \end{array}$$

The stoichiometric matrix ***S*** is a *D* × *r* dimensional matrix. Its entries *s_ij_* = *u_ij_* − *q_ij_* describe the net effect of reaction *j* to species *X_i_*. In terms of ODEs, the systems would read as

$$\frac{d}{dt}x(t;\;\theta ,\;x_{0})=S\;\upsilon \;(x(t;\;\theta ,\;x_{0}),\;\theta ),\;x(0,\;\theta ,\;x_{0})=x_{0}$$

with a rate law *υ* = (*υ*_1_, …, *υ* *_r_*)^T^ describing the speed of the reactions and initial concentrations *x*_0_.

Stochastic modelling is important in systems with small numbers of molecules, in which stochastic fluctuations can influence the systems’ behaviour [24]. It focuses on single species and considers each reaction explicitly. The temporal order of the reactions and the waiting times are stochastic quantities depending on the state of the system and the rate laws. For simulating a stochastic time course, the Gillespie algorithm [2] is the method of choice. It is an iterative algorithm, simulating reaction event after reaction event, using functions of random numbers to determine the time step and the reaction. There exists a number of implementations [3] of the Gillespie algorithm. The resulting time course is a discrete state continuous time Markov jump process, see also [25] for details.

Stochastic modelling can show dynamic behaviour which cannot be seen with ODE modelling: stochasticity can, for example, introduce bistability in genetic toggle switches which have a steady state in ODE modelling [26]. The structure of calcium oscillations may change qualitatively from ODE to stochastic modelling [27]. Furthermore, intrinsic stochasticity may provide information, for example, in form of reactivity, which allows to solve identifiability problems [4]. This shows the importance of stochastic modelling and the need for suited parameter estimation methods for stochastic models.

### 2.2 Approximation

As mentioned in Section 1, the parameter estimation is based on maximising a likelihood function that is a product of transition probabilities. An accurate calculation of these transition probabilities is very time‐consuming. To overcome this problem, we need to find an approximate way to estimate the transition probabilities that is fast enough to be applicable in realistic size systems and still accurate enough to capture intrinsic stochastic features. The original MSS method suggests a rather simple approximation with a normal distribution and constant variance [4]. Although this approach has been shown to work well in several cases, we expect that a more accurate approximation will lead to more accurate estimation results or allow parameter estimation for a larger set of models. Therefore we extend the approximation by taking into account the variability of the variances of the transition probabilities.

Let the system be in a state *ν_i_*_−1_ at time *t_i_*_−1_. We approximate the distribution of *ν* at time *t_i_*_−1_ + Δ*t* by

(1) $$\begin{array}{rl} & \nu_{i}∼N(\mu ,\;Cov)\\ & \text{with}\;\;\mu =x(\Delta t;\;\theta ,\;\nu_{i-1})\;(\text{ODE}\;\;\text{solution}\;\;\text{on}\;\;\\ & \;[t_{i-1},\;t_{i}]\;\;\text{with}\;\;\text{initial}\;\;\text{value}\;\;\nu_{i-1})\\ & \text{and}\;\;Cov=\sum (\Delta t;\;\theta )\;(\text{LNA}\;\;\text{approximation}\;\;\text{on}\;\;\\ & \;[t_{i-1},\;t_{i}]\;\;\text{with}\;\;\text{initial}\;\;\text{value}\;\;0_{D\times D}) \end{array}$$

On the time interval Δ*t*, the mean *μ* is the ODE solution of the system and the variance ∑ is calculated by an LNA. **0** *_D_*_×_ *_D_* stands for a *D* × *D* matrix with all entries equal zero. The LNA can be calculated with only the knowledge of the stoichiometric matrix ***S*** and the rate law vector *υ*

(2) $$\begin{array}{rl} \frac{d}{dt}\sum (t;\;\theta ) & =J(x(\Delta t;\;\theta ,\;\nu_{i-1}),\;\theta )\sum (t;\;\theta )\\ & \;+\sum (t;\;\theta )J(x(\Delta t;\;\theta ,\;\nu_{i-1}),\;\theta ){\;}^{T}\\ & \;+\Omega^{-1}D(x(\Delta t;\;\theta ,\;\nu_{i-1}),\;\theta ),\;\sum (0,\;\theta )=0_{D\times D}\\ & \text{with}\;\;J(x,\;\theta )=S\frac{d}{dx}\upsilon \;(x,\;\theta )\\ & \text{and}\;\;D=(D_{ij})\;\;\text{with}\;\;D_{ij}(x,\;\theta )=\sum_{k=1}^{R}S_{ik}S_{\;jk}\upsilon_{k}(x,\;\theta ) \end{array}$$

and the volume **Ω**. Details and derivation can be found in [28]. See also [29] ((6) and Appendix) and [30, 31, 32], which discuss software tools for calculating LNAs.

The approximation differs from the approach used in the old MSS method [4], in that the covariance is not kept constant over the trajectory, but is calculated by an LNA on each time interval.

Although the validity of the LNA depends on the model and its parametrisation, and thus cannot be guaranteed a priori, the LNA is much more likely to hold on the intervals between measurements than for the whole time series. In many cases LNAs are applied to approximate stochastic systems from the first until the last observation. This means that the stochastic dynamics must fulfil the condition from the beginning until the end. The point where the LNA typically fails is when the probability distribution of the state variables deviate significantly from a normal distribution, that is, when the system cannot be considered to behave such as the deterministically simulated model plus some noise. Unfortunately, this includes more or less exactly those cases where we have to choose a stochastic modelling approach because an observed effect is not explained by a deterministic model. Since the error of the LNA grows over time, its validity conditions can be fulfilled much easier if we only apply it on the much shorter time intervals between measurements. Note that in this use case, we only require the LNA to hold locally on each of the intervals since we only use it to calculate the transition probabilities for each interval, from which we determine a global likelihood function. We do not combine the local LNAs to one global approximation of the system's dynamics, and therefore we do not have to test how the approximation errors of each of the LNAs combine to a global error.

Additionally, this approximation can not only be used for parameter estimation, but also for simulating trajectories. Choose a set of time points $t_{0}<\cdots <t_{n}$ and an initial state *ν*_0_. Then, draw random numbers according to (1) to iteratively calculate the values for the system states *ν*_1_, …, *ν_n_*. Rounding the *ν_i_* to integers and defining the process’ state to be *ν_i_*_−1_ on [*t_i_*_−1_, *t_i_*) leads to a trajectory that is a continuous time discrete state Markov jump process.

Naturally, the question arises how to check whether the approximation holds for a specific system under consideration. We suggest a fast and intuitive check, discuss the formal conditions and propose a calculation that can be carried out a posteriori showing how good the approximation worked

- Since the approximation can also be used for an approximative simulation, we can perform a first check by visually comparing the approximate trajectory with one obtained from an exact simulation method (such as the Gillespie algorithm). Although this will not tell us about the quantitative accuracy of the approximation, it can tell us if the qualitative features of the trajectory, and especially the stochastic effects that lead us to use a stochastic modelling approach, are conserved.
- The theoretical condition for the validity of the approximation on the interval [*t_i_*_−1_, *t_i_*] is the condition of an LNA on this interval. The theoretical condition for the validity first uses a variable transformation to write the CME in terms of concentrations and volumes. Next, the resulting equation is expanded in inverse powers of the volume (similar to a Taylor expansion). If higher‐order terms are small and can be neglected, the LNA holds and one can calculate the remaining terms (after cutting off the higher‐order terms) as described above. Details for the expansion and its derivation can be found in [28, 30].
- Given time series data and a parameter, it is possible to reconstruct the random number for each interval that is necessary to obtain the data. Using the notation from (1), this is $r_{i}=\sum^{-1/2}(\nu_{i}-x)$, with *ν_i_* being the data point at *t_i_*. Owing to the multiplication with the square root of the inverse of its corresponding covariance matrix *r_i_* should be an *N*(0*_D_*, **1** *_D_*_×_ *_D_*)‐distributed random number if the approximation holds, where **1** *_D_*_×_ *_D_* stands for a *D* × *D*‐matrix with diagonal entries 1. Checking whether the random numbers *r*_1_, …, *r_n_* indeed follow this distribution, can give information on the accuracy of the approximation (details in Section 2.6).

### 2.3 Extended objective function

Given experimental data as input, parameter estimation uses numerical techniques to calculate a parameter which calibrates the model to the data.

Denote the data as *ν* = (*ν*_0_, …, *ν_n_*), measured at time points *t*_0_, …, *t_n_*. The likelihood function *L*(*ν*, *θ*) gives the probability to observe the data *ν* for a given parameter *θ*. With the help of the approximation in the previous section, it is possible to derive a formula for the likelihood function. Owing to the Markov property of biochemical reaction networks [25], the likelihood function factorises into the product of the transition probabilities

$$L(\nu ,\;\theta )=\prod_{i=1}^{n}p(\nu_{i};\;\nu_{i-1},\;\Delta_{i},\;\theta )$$

where Δ*_i_* = *t_i_* − *t_i−_*_1_ and *p*(*ν_i_*;*ν_i_*_−1_, Δ*_i_*, *θ*) is the probability for a transition from state *ν_i−_*_1_ at time *t_i−_*_1_ to state *ν_i_* at time *t_i_* given the parameter *θ*. This transition probability will be approximated with a normal distribution as in (1)

$$\begin{array}{rl} & p(\nu_{i};\;\nu_{i-1},\;\Delta_{i},\;\theta )\approx \text{PDF}\left(N\left(x(\Delta_{i};\;\theta ,\;\nu_{i-1}),\;\sum (\Delta_{i};\;\theta )\right),\;\nu_{i}\right)\\ & \text{with}\;\;x\;\;\text{and}\;\;\sum \;\;\text{as}\;\;\text{in}\;\;\text{equation}\;\;(1) \end{array}$$

where PDF(dist, *y*) stands for probability density function (PDF) of the distribution ‘dist’ at *y*. Using the negative logarithm of the likelihood function, this leads to the extended MSS objective function

(3) $$\begin{array}{rl} & F_{\text{MSS}}(\nu ,\;\theta )=-\sum_{i=1}^{n}\text{log}\left(\text{PDF}\left(N\left(x(\Delta_{i};\;\theta ,\;\nu_{i-1}),\;\sum (\Delta_{i};\;\theta )\right),\;\nu_{i}\right)\right)\\ & \text{with}\;\frac{d}{dt}x(t;\;\theta ,\;\nu_{i-1})=S\upsilon \left(x(t;\;\theta ,\;\nu_{i-1}),\;\theta \right),\;x(0;\;\theta ,\;\nu_{i-1})=\nu_{i-1}\\ & \frac{d}{dt}\sum (t;\;\theta )=J(x(t;\;\theta ,\;\nu_{i-1}),\;\theta )\sum (t;\;\theta )\\ & \;\;\;\;\;+\sum (t;\;\theta )J(x(t;\;\theta ,\;\nu_{i-1}),\;\theta ){\;}^{T}\\ & \;\;\;\;\;+\Omega^{-1}D(x(t;\;\theta ,\;\nu_{i-1}),\;\theta ),\;\sum (0;\;\theta )=0_{D\times D} \end{array}$$

As mentioned in Section 1, from a numerical point of view the method is motivated by the multiple shooting method of [33] that performs the integration on the so‐called multiple shooting subintervals. Equality constraints between the end point of a previous interval and initial value variables on the subintervals lead to a continuous trajectory. This method shows beneficial behaviour for parameter estimation in ODE systems [34, 35]. The MSS method is similar to the cited method as it also uses a multiple shooting approach, but it differs by not using the equality constraints. In a statistical setting the method is a Gaussian, non‐linear, first‐order autoregressive time series model. An ODE integration initialised with the previous observation is used as the mean of the next interval; the conditional variance is obtained from an LNA on the intervals between succeeding time points of measurement.

A maximum‐likelihood parameter estimate can be calculated by maximising the likelihood function or equivalently minimising the negative log likelihood function. Using the approximation, a parameter estimate $\hat{\theta }$ can be determined by

$$\hat{\theta }={\text{argmin}}_{\theta }F_{\text{MSS}}(\nu ,\;\theta )$$

### 2.4 Partially observed data

In typical experimental setups, it is not possible to obtain measurements for every species taking part in the reactions. Assume that the first *d* species of the system are observable and denote their measurements with *ν* ^obs^ and denote the unobserved or hidden components with *ν* ^hid^ so that *ν* = (*ν* ^obs^, *ν* ^hid^) and the data $\nu^{\text{obs}}=\left(\nu_{0}^{\text{obs}},\;\ldots ,\;\nu_{n}^{\text{obs}}\right)$ is measured at time points *t*_0_, …, *t_n_*.

Include the unobserved species at time *t*_0_, $\nu_{0}^{\text{hid}}$, into the optimisation vector. Owing to the approximation, the distribution at time point *t*_1_ is a normal distribution. As a very simple state estimate for the unobserved species $\nu_{1}^{\text{hid}}$, we choose the most likely value from this distribution, which is the mean. It is in this case calculated by the ODE solution on the time interval. More generally, estimate the unobserved state ${\hat{\nu }}_{i}^{\text{hid}}$ at time *t_i_* for *i* = 1, …, *n* − 1 by

$$\begin{array}{rl} & {\hat{\nu }}_{i}^{\text{hid}}=x(\Delta_{i},\;\theta ,\;\nu_{i-1}){\;}^{\text{hid}}\\ & \frac{d}{dt}x(t;\;\theta ,\;\nu_{i-1})=S\upsilon \;\left(x(t;\;\theta ,\;\nu_{i-1}),\;\theta \right),\;\\ & \;x(0;\;\theta ,\;\nu_{i-1})=\left(\nu_{i-1}^{\text{obs}},\;{\hat{\nu }}_{i-1}^{\text{hid}}\right) \end{array}$$

In this case the objective function reads as

(4) $$\begin{array}{rl} & F_{\text{MSS}}(\nu ,\;\theta ,\;\nu_{0}^{\text{hid}})\\ & =-\sum_{i=1}^{n}\text{log}\left(\text{PDF}\left(N\left(x(\Delta_{i};\;\theta ,\;{\tilde{\nu }}_{i-1}){\;}^{\text{obs}},\;\sum (\Delta_{i};\;\theta ){\;}^{\text{obs}}\right),\;\nu_{i}^{\text{obs}}\right)\right)\\ & {\tilde{\nu }}_{i-1}=\left\{\begin{array}{cc} (\nu_{0}^{\text{obs}},\;\nu_{0}^{\text{hid}}),\; & i-1=0\\ \left(\nu_{i-1}^{\text{obs}},\;x(\Delta_{i-1};\;\theta ,\;{\tilde{\nu }}_{i-2}){\;}^{\text{hid}}\right), & i-1>0 \end{array}\right.\\ & \text{with}\frac{d}{dt}x(t;\;\theta ,\;{\tilde{\nu }}_{i-1})=S\upsilon \;\left(x(t,\;\theta ,\;{\tilde{\nu }}_{i-1}),\;\theta \right),\;\\ & \;x(0;\;\theta ,\;{\tilde{\nu }}_{i-1})={\tilde{\nu }}_{i-1}\\ & \frac{d}{dt}\sum (t;\;\theta )=J(x(t;\;\theta ,\;{\tilde{\nu }}_{i-1}),\;\theta )\sum (t;\;\theta )\\ & \;+\sum (t;\;\theta )J(x(t;\;\theta ,\;{\tilde{\nu }}_{i-1}),\;\theta ){\;}^{T}\\ & \;+\Omega^{-1}D(x(t;\;\theta ,\;{\tilde{\nu }}_{i-1}),\;\theta ),\;\sum (0;\;\theta )=0_{D\times D} \end{array}$$

The estimator in the partially observed scenario is determined by an optimisation over the parameter *θ* and the unobserved initial states $\nu_{0}^{\text{hid}}$

$$\left(\hat{\theta },\;{\hat{\nu }}_{0}^{\text{hid}}\right)={\text{argmin}}_{\left(\theta ,\;\nu_{0}^{\text{hid}}\right)}F_{\text{MSS}}(\nu ,\;\theta ,\;\nu_{0}^{\text{hid}})$$

It might be noted that the fully observed case is a subcase of this scenario with *ν* = *ν* ^obs^ and *ν* ^hid^ = {}. Therefore in the following the notation of the partially observed case will be used. Our choice to estimate the unobserved state variables solely from deterministic simulation over the preceding time interval is guided by the desire to provide a very simple objective function. A more accurate estimate would take into account the correlation between unobserved and observed species, as predicted by the LNA. In principle, the unknown state variables could even be estimated including information from future measurements, for example, by using Kalman smoothing techniques [36]. This would, however, run contrary to the underlying principle for our approach that the intervals between measurement points are treated independently. And our goal for this paper is to show that even a simple state estimate can serve well for parameter estimation.

### 2.5 Measurement noise

Measurement noise is assumed to be normally distributed with zero mean. Furthermore, it is assumed to be independent of the intrinsic stochasticity of the process and inter‐temporally independent, which means that the measurement errors of two time points are independent. Apart of that the *d* × *d* covariance matrix $\left(\sum^{\text{meas}}\right)$ of the measurement error at a certain time point may account for correlated errors between the species. More formally the measurement error at time point *t_i_* is distributed with $N(0_{d},\;\sum^{\text{meas}})$. In this case, the first line of (4) extends to

$$\begin{array}{rl} & F_{\text{MSS}}(\nu ,\;\theta ,\;\nu_{0}^{\text{hid}})=\\ & \;-\text{log}\sum_{i=1}^{n}(\text{PDF}\left(N\left(\begin{array}{c} x(\Delta_{i};\;\theta ,\;\nu_{i-1}){\;}^{\text{obs}},\;\sum (\Delta_{i};\;\theta ){\;}^{\text{obs}}+\sum^{\text{meas}} \end{array}\right),\;\nu_{i}^{\text{obs}}\right)) \end{array}$$

Assuming that the noise model is given (e.g. from knowledge about the experimental setup) and has zero mean, the observed data point can serve as a simple state estimate. In principle, a better estimate could be calculated by also taking into account the prediction of the model and measurements of other variables as well as measurements at other time points. As mentioned in the previous section, the focus of this paper is to show that even this very simple approach allows for good parameter estimation results.

### 2.6 Accuracy of the approximation

For given time series measurements and parameter values, it is possible to test how well the approximation works: instead of calculating the sum in the objective function *F*_MSS_, calculate the following values

(5) $$\begin{array}{rl} & r_{i}^{\text{obs}}={\left(\sum (\Delta_{i};\;\theta ){\;}^{\text{obs}}+\sum^{\text{meas}}\right)}^{-1/2}\left(x(\Delta_{i};\;\theta ,\;{\tilde{\nu }}_{i-1}){\;}^{\text{obs}}-\nu_{i}^{\text{obs}}\right) \end{array}$$

for *i* = 1, …, *n* with the solution of the ODE system for *x* and ∑ as in (4). Taking into account (1), it follows that $r_{i}∼N(0_{d},\;1_{d\times d})$, where **1** *_d_*_×_ *_d_* denotes a *d* × *d*‐matrix with diagonal entries one. This means that it is enough to test whether $r^{\left(k\right)}=\left(r_{1}^{\left(k\right)},\;\ldots ,\;r_{n}^{\left(k\right)}\right)∼N(0,\;1)$ for *k* = 1, …, *d*. This can be used to check how well the approximation works. Note that there are *n* + 1 measurements, but only *n* transition probabilities, and therefore only *n* numbers *r_i_* as well.

### 2.7 Optimisation

The objective function is completely deterministic, which means that for its evaluation no stochastic simulations have to be performed. The optimisation problem can therefore be solved using gradient‐based methods. Of course, it is as well possible to use global optimisation techniques or Bayesian approaches.

## 3 Results

### 3.1 Immigration‐death model

The first example is an immigration‐death model

$$\begin{array}{rl} & Ø\overset{\theta_{1}}{⟶}\;\;X\\ & X\overset{\theta_{2}}{⟶}\;\;Ø \end{array}$$

where *X* is the substance and *θ*_1_, *θ*_2_ are parameters and a rate law *v* = (*θ*_1_, *θ*_2_ *x*). The stoichiometric matrix is ***S*** = (1, −1) and the system in ODE representation reads as

$$\frac{d}{dt}x(t;\;\theta ,\;x_{0})=\theta_{1}-\theta_{2}x,\;x(0;\;\theta ,\;x_{0})=x_{0}$$

#### 3.1.1 Considered scenarios

Several parametrisations and choices of time points for measurements are considered. The scenarios have been selected according to the choice of [8] to be able to compare the accuracy of the estimation results. The initial value is chosen in a way that the system is in the deterministic steady state, *x*_0_ = *θ*_1_/*θ*_2_.

#### 3.1.2 Design of simulation study

One difficulty when evaluating the quality of a parameter estimate for a stochastic process is that the observed data (in our case the test data generated from the stochastic process) is itself random. This means that even when a method is deterministic, and so gives the same estimate when run repeatedly on the same time series, it will result in different estimates if applied to different realisations of the same process. Therefore it is not possible to estimate the accuracy of a method by just looking at the results based on one set of observations. Any deviation between true and estimated parameter values could either be the result of a random deviation of the data or of a bias in the method. To resolve this problem, the estimation process was performed 100 times with 100 different data sets for each scenario. Note that this is only needed to ‘test’ the method; it is still possible to ‘apply’ it with a single time series. The data sets were created by running 100 simulations with the Gillespie algorithm [2] in the software COPASI [37] Version 4.11‐65. For each time series, an estimation is performed with the extended MSS method. For the resulting 100 parameter estimates ${\hat{\theta }}^{\left(i\right)}$, *i* = 1, …, 100 the average $\text{av}=(1/100)\sum_{i=1}^{100}{\hat{\theta }}^{\left(i\right)}$ and the average relative error (ARE) $\text{ARE}=(1/100)\sum_{i=1}^{100}\left(|\;{\hat{\theta }}^{\left(i\right)}-\theta^{\left(0\right)}|/\theta^{\left(0\right)}\right)$, with *θ* ^(0)^ denoting the true parameter, are displayed in Table 1.

**Table 1:** Statistics of the estimation results for immigration‐death model

*θ* ^(0)^ (#Δ*t*)	(0.6, 0.03)	(0.6, 0.06)	(0.6, 0.1)	(0.1, 0.03)	(0.2, 0.03)
(21 2)	av:(0.59, 0.03)	av:(0.62, 0.06)	av:(0.64,0.11)	av:(0.106, 0.032)	av:(0.2,0.027)
	ARE:(33%, 34%)	ARE:(31%, 34%)	ARE:(28%, 34%)	ARE:(46%, 47%)	ARE:(35%, 32%)
	*w*:(0.61, 0.03)	*w* = (0.36, 0.041)	*w* = (0.47, 0.101)	*w* = (0.167, 0.035)	*w* = (0.28, 0.02)
	rank 1	rank 68	rank 14	rank 51	rank 57
(51 2)	av:(0.6, 0.03)	av:(0.61, 0.063)	av:(0.6, 0.103)	av:(0.09, 0.033)	av:(0.21, 0.033)
	ARE:(16%, 16%)	ARE:(21%, 20%)	ARE:(24%, 24%)	ARE:(28%, 37%)	ARE:(27%, 25%)
	*w*:(0.78, 0.03)	*w* = (0.75, 0.077)	*w*:(0.63, 0.12)	*w*:(0.074, 0.032)	*w*:(0.15, 0.029)
	rank 59	rank 72	rank 28	rank 21	rank 26
(21 5)	av:(0.62, 0.031)	av:(0.61, 0.063)	av:(0.67, 0.108)	av:(0.1, 0.032)	av:(0.21, 0.034)
	ARE:(31%, 31%)	ARE:(30%, 30%)	ARE:(35%, 31%)	ARE:(37%, 28%)	ARE:(36%, 35%)
	*w*:(0.51, 0.026)	*w*:(0.67, 0.082)	w:(0.69, 0.12)	*w*:(0.092, 0.026)	*w*:(0.21, 0.028)
	rank 21	rank 49	rank 37	rank 10	rank 6
(101 10)	av = (0.62, 0.031)	av:(0.63, 0.063)	av:(0.76, 0.126)	av:(0.11, 0.032)	av:(0.20, 0.031)
	ARE:(13%, 15%)	ARE:(16%, 16%)	ARE:(40%, 39%)	ARE:(16%, 17%)	ARE:(14%, 14%)
	*w*:(0.51, 0.026)	*w*:(0.42, 0.04)	*w*:(0.43, 0.067)	*w*:(0.094, 0.024)	*w*:(0.175, 0.026)
	rank 56	rank 91	rank 73	rank 47	rank 57

This table shows the performance of the extended MSS method on an immigration‐death model and compares it with results calculated by Wang *et al.* [8]. Different parametrisation are considered (columns) as well as different measurement scenarios (rows). *θ* ^(0)^ stands for the true parameter, # stands for number of measurement points and Δ*t* stands for the time interval between two succeeding observations. For each of the 20 scenarios, 100 simulated time series are calculated with the Gillespie algorithm [2] within COPASI [37] Version 4.11‐65. For each of the time series, an estimator ${\hat{\theta }}^{\left(i\right)}$ is calculated. The table shows
the average of the estimators: $\text{av}=\frac{1}{100}\sum_{i=1}^{100}{\hat{\theta }}^{\left(i\right)}$
their ARE: $\text{ARE}=\frac{1}{100}\sum_{i=1}^{100}\frac{\left|{\hat{\theta }}^{\left(i\right)}-\theta^{\left(0\right)}\right|}{\theta^{\left(0\right)}}$

*w* is the estimate of one time series shown by Wang e*t al.* [8] The rank of *w* among the 100 estimates of this paper with respect to relative error (details on how this rank is calculated can be found in Sections 3.1.2). The table shows that the extended MSS method estimates the parameters with acceptably small relative error for all considered scenarios. In comparison with the reversible jump method by Wang *et al.* [8], the relative error is not significantly larger, which shows that the high computational speed of the method does not lead to a loss of accuracy.

For comparison Table 1 includes the estimate of [8]. In [8] no simulation study is performed and hence only one estimate is provided. Thus we can only state how this one result ranks in comparison with our 100 results. The relative error of this estimate is calculated as well as the 100 relative errors of our estimates. The 101 relative errors are sorted by size (from low to high) and the rank of [8]'s estimate is recorded. A rank 1 for most of the scenarios would indicate that the method of [8] is clearly better, a rank 101 that it is clearly worst, and ranks in between that the accuracy is comparable.

The optimisation has been performed with the FindMinimum routine implemented in the software Mathematica [38] Version 8.0.4.0.

#### 3.1.3 Observations

**Fig. 2:**
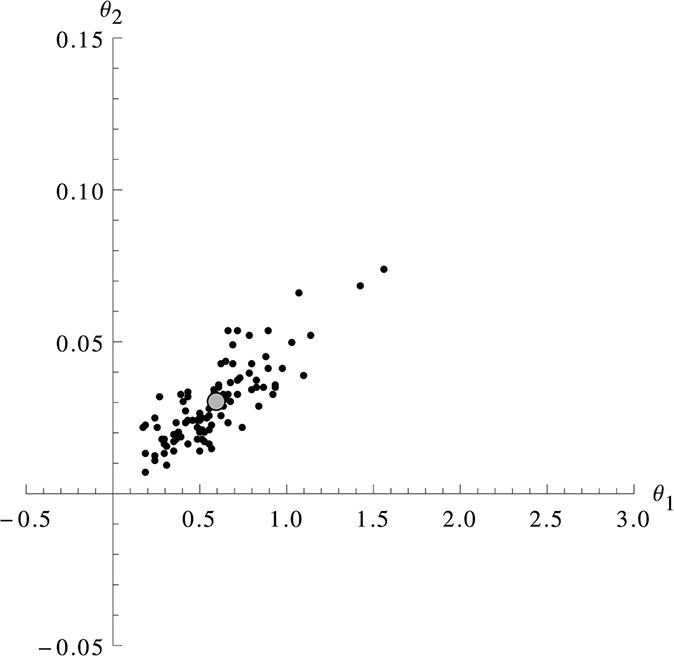
Estimates for an immigration‐death scenario For the scenario of 21 measurements with Δ*t* = 2 (table 1, first row, first column) and *θ* ^(0)^ = (0.6,0.03) the graphic shows the 100 estimates (black dots) and the true parameter value *θ* ^(0)^ (big grey dot). Using a deterministic model, only the ratio but not the absolute value of the parameters is identifiable. Using the MSS method, one can see that not only the ratio but also the absolute value is identifiable, however, with a slightly worse accuracy.

The estimations of the simulation study show that the extended MSS method is able to estimate both parameters for all considered scenarios. As a reference for the accuracy of the estimate, we compare our 100 results to the one result for each scenario that is given in [8]. In each but one case, the result of [8] falls well into the range of our results. From this, we conclude that our method shows comparable accuracy while being computationally less demanding. Fig. 2 shows a plot of the estimation of the first row and first column of Table 1 in a coordinate system, illustrating how the estimates spread around the true parameter. Both parameters are identifiable (there is a limited range of values for them), but the stretched form of the cloud indicates that the ratio of the parameters can be identified more accurately. This is expected since in the case of a deterministic model the ratio would be the only identifiable value while the two separate parameters would be completely unidentifiable.

**Fig. 3:**
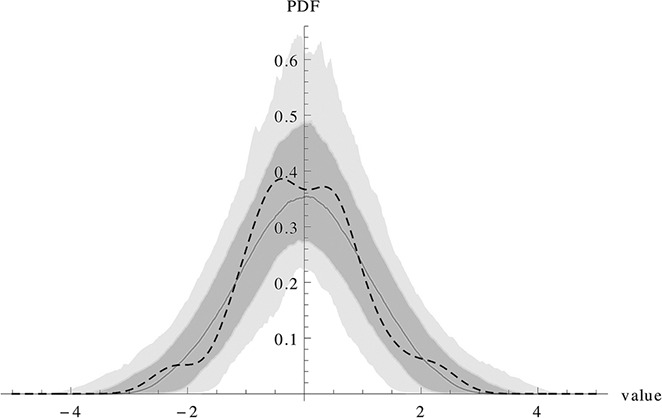
Checking the approximation for one time course This figure checks how well the approximation works for a time course of the Immigration‐Death model. As the data sets contain 21 measurements, 21‐1 *N*(0, 1) random variables are drawn and a density estimation is performed with the SmoothKernelDistribution function in Mathematica 9 [39]. This action is repeated 1000 times. The solid line shows the mean of the 1000 densities and the yellow area fills the area from the 10%‐quantile to the 90%‐quantile, the grey area from the 1%‐quantile to the 99%‐quantile. The graphic shows an example of the ID11 scenario. The dashed line shows the density estimate of the (*r*_1_,…,*r*_20_) of equation (5). One can see that these numbers are compatible with the assumption of a *N*(0, 1) distribution which is a condition for the validity of the approximation.

Fig. 3 shows the result of testing the validity of the approximation using the concept described in Section 2.6. The basic idea is that for each time step the deviation of the data value from the result of a deterministic simulation should be normally distributed with variance as calculated from the LNA. The result is only shown for one time course, but would hold for most other time courses as well.

Only the case of parameter values (0.1, 0.03) leads to situations in which for a few time series no convergence could be achieved, namely 5 of 100 for the first measurement scenario, 1 of 100 for the second, 2 of 100 for the third and none for the fourth. Examining the time series for which no convergence could be achieved shows that they are untypical in that they just decay from the starting value to zero, indicating that no or very few influx events actually occurred during the observation time. It is understandable that the rate of the influx reaction (i.e. the propensity of an influx event) cannot be estimated if the reaction does not occur during the observation time. Checking the validity of the approximation, using the concept described above, shows that the approximation does not hold in the cases where convergence could not be achieved.

An increase of the number of measurement points with the same inter‐sample distance (row 1 to row 2 of Table 1) shows that the accuracy increases. Varying the inter‐sample distance and keeping the number of observations fixed (row 1 and row 3 of Table 1) does not show a clear effect as for columns 1, 2 and 4 the accuracy is improved, but not for columns 3 and 5. The reason is that the optimal inter‐sample distance depends on the parameter. Methods to choose an inter‐sample distance before doing any experiments are called optimal design methods and are ongoing research. Note that for none of the scenarios the parameters would be identifiable using a conventional deterministic least‐squares approach.

Comparing the results of the extended MSS method to the original MSS approach with constant variances (results not shown), shows that the old approach is only able to estimate the parameters correctly in the scenarios with 100 observations and parameters *θ* = (0.6, 0.03) or *θ* = (0.6, 0.06) with an ARE of (31%, 32%) and (21%, 24%), which is still higher than with the extended MSS. The other scenarios are time courses in which only very few reactions happen between two time points of measurement. These are problematic for the old MSS as indicated in [4]. With the improved method now all scenarios are tractable.

The immigration‐death model is one of the few stochastic models in which it is possible to solve the CME analytically, which allows for a calculation of exact estimates without approximation. This means that the remaining error of the estimation is because of the stochasticity of the time series data, rather than because of approximations in the estimation process. Comparing these results to the extended MSS approach for the scenario of 101 measurements and parameter (0.6, 0.06), shows that the ARE of the extended MSS approach (16%, 16%) is only slightly higher than the ARE of the exact method (15%, 16%). This shows that the extended MSS method is able to extract almost all information out of the data.

### 3.2 Prokaryotic auto‐regulatory network

The second example has also been investigated by Wang *et al.* [8] and is an example of gene regulation

$$\begin{array}{rl} \text{DNA}+P_{2} & \overset{\theta_{1}}{⟶}\;\;\text{DNA}.P_{2}\\ \text{DNA}.P_{2} & \overset{\theta_{2}}{⟶}\;\;\text{DNA}+P_{2}\\ \text{DNA} & \overset{\theta_{3}}{⟶}\;\;\text{DNA}+m\text{RNA}\\ m\text{RNA} & \overset{\theta_{4}}{⟶}\;\;Ø\\ 2P & \overset{\theta_{5}}{⟶}\;\;P_{2}\\ P_{2} & \overset{\theta_{6}}{⟶}\;\;2P\\ m\text{RNA} & \overset{\theta_{7}}{⟶}\;\;m\text{RNA}+P\\ P & \overset{\theta_{8}}{⟶}\;\;Ø \end{array}$$

where DNA represents promoter sequences, *P* proteins, *P*_2_ protein dimmers, *m*RNA messenger RNA and *θ*_1_, …, *θ*_8_ parameters. All reactions are modelled with mass action as kinetic law. An representation in ODEs reads as

$$\begin{array}{rl} & \frac{d}{dt}\text{DNA}(t)=-\theta_{1}\text{DNA}(t)P_{2}(t)+\theta_{2}\text{DNA}.P_{2}(t),\text{DNA}(0)={\text{DNA}}_{0},\\ & \frac{d}{dt}P_{2}(t)=-\theta_{1}\text{DNA}(t)P_{2}(t)+\theta_{2}\text{DNA}.P_{2}(t)\\ & \;\;\;\;+\theta_{5}P(t){\;}^{2}-\theta_{6}P_{2}(t),\;P_{2}(0)={P_{2}}_{0},\\ & \frac{d}{dt}\text{DNA}.P_{2}(t)=\theta_{1}\text{DNA}(t)P_{2}(t)-\theta_{2}\text{DNA}.P_{2}(t),\;\\ & \;\text{DNA}.P_{2}(0)={\text{DNA}.P_{2}}_{0}\;,\\ & \frac{d}{dt}m\text{RNA}(t)=\theta_{3}\text{DNA}(t)-\theta_{4}m\text{RNA}(t),\;m\text{RNA}(0)=m{\text{RNA}}_{0}\\ & \frac{d}{dt}P(t)=-2\theta_{5}P(t){\;}^{2}+2\theta_{6}P_{2}(t)+\theta_{7}m\text{RNA}(t)-\theta_{8}P(t),\;P(0)=P_{0} \end{array}$$

As in [8], the true parameter value is chosen as *θ* ^(0)^ = (0.1, 0.7, 0.35, 0.3, 0.1, 0.9, 0.2, 0.1) and the initial conditions *P*_2 0_ = 6, *m*RNA_0_ = 8 and *P*_0_ = 25. Other initial values are varied within the study and displayed separately in the tables. DNA*_t_* = DNA(*t*) + DNA.*P*_2_(*t*) is modelled a conserved quantity.

#### 3.2.1 Considered scenarios

Different choices of measurement time points are considered. All scenarios are considered in full observation and in partial observation. For the partially observable case, measurements are taken for *m*RNA, *P* and *P*_2_. The total amount DNA*_t_* is assumed to be known as well. Furthermore, two different values for DNA*_t_* are considered.

#### 3.2.2 Design of simulation study

For each scenario, 100 simulations are performed as described above for the immigration‐death model to generate the pseudo‐data. For each of the 100 time series, an estimation is performed with the extended MSS method. For the resulting 100 parameter estimates ${\hat{\theta }}^{\left(i\right)}$, *i* = 1, …, 100 the average $\text{av}=(1/100)\sum_{i=1}^{100}{\hat{\theta }}^{\left(i\right)}$, the ARE $\text{ARE}=(1/100)\sum_{i=1}^{100}\left(\;|{\hat{\theta }}^{\left(i\right)}-\;\theta^{\left(0\right)}|/\theta^{\left(0\right)}\right)$ and the median of the 100 relative errors are displayed in Tables 2 and 3.

**Table 2:** Statistics of the estimation results for an auto‐regulatory gene network – fully observed

		*θ*_1_	*θ*_2_	*θ*_3_	*θ*_4_	*θ*_5_	*θ*_6_	*θ*_7_	*θ*_8_	
(# Δ*t*)		0.1	0.7	0.35	0.3	0.1	0.9	0.2	0.1	rank
(50 1)	av	0.294	2.051	0.367	0.323	0.405	3.624	0.197	0.1	div 18
DNA*_t_* = 10	ARE	222%	226%	20%	20%	344%	341%	18%	18%	
	medRE	31%	32%	16%	17%	47%	44%	15%	17%	
	*w*1	0.114	0.81	0.346	0.229	0.051	0.418	0.221	0.074	rank 21
	*w*2	0.094	0.72	0.435	0.344	0.052	0.485	0.265	0.119	rank 21
(100 0.5)	av	0.111	0.753	0.351	0.299	0.257	2.337	0.194	0.097	div 4
DNA*_t_* = 10	ARE	21%	19%	18%	16%	185%	184%	17%	17%	
	medRE	13%	13%	17%	12%	28%	26%	13%	12%	
	*w*1	0.113	0.82	0.408	0.321	0.075	0.75	0.226	0.095	rank 20
	*w*2	0.113	0.71	0.276	0.253	0.086	0.77	0.223	0.1	rank 11
(500 0.1)	av	0.101	0.696	0.349	0.305	0.094	0.905	0.202	0.101	div 0
DNA*_t_* = 10	ARE	8%	7%	13%	12%	9%	9%	14%	15%	
	medRE	7%	6%	10%	11%	8%	8%	11%	12%	
	*w*1	0.079	0.74	0.349	0.286	0.101	0.86	0.183	0.094	rank 10
(50 1)	av	0.421	2.275	0.36	0.327	0.394	3.358	0.197	0.101	div 22
DNA*_t_* = 2	ARE	340%	246%	26%	25%	336%	309%	35%	16%	
	medRE	21%	24%	18%	19%	36%	30%	31%	13%	
	*w*1	0.095	0.42	0.321	0.277	0.10	0.73	0.235	0.104	rank 2
	*w*2	0.097	0.9	0.35	0.335	0.079	0.92	0.312	0.12	rank 8
(100 0.5)	av	0.105	0.687	0.359	0.309	0.298	2.552	0.199	0.1	div 8
DNA*_t_* = 2	ARE	23%	23%	29%	18%	220%	203%	25%	17%	
	medRE	21%	20%	26%	15%	25%	25%	20%	14%	
	*w*1	0.12	0.4	0.52	0.38	0.092	0.998	0.215	0.081	rank 42
	*w*2	0.116	0.96	0.41	0.41	0.101	1.01	0.144	0.094	rank 27
(500 0.1)	av	0.107	0.623	0.302	0.315	0.092	0.895	0.215	0.096	div 8
DNA*_t_* = 2	ARE	17%	23%	32%	25%	11%	7%	34%	16%	
	medRE	14%	19%	27%	18%	10%	5%	28%	16%	
	*w*1	0.052	0.91	0.277	0.35	0.128	0.93	0.137	0.075	rank 73

This table shows the performance of the extended MSS method on an auto‐regulatory gene network model and compares it with results calculated by Wang *et al.* [8]. Different measurement scenarios are considered as well as different amounts of DNA*_t_*. The initial values are for DNA*_t_* = 10: DNA_0_ = 6, DNA.*P*_2_(0) = 4 as well as for DNA*_t_* = 2: DNA_0_ = 2, DNA.*P*_2_(0) = 0. *θ* ^(0)^ stands for the true parameter, # stands for number of measurement points and Δ*t* stands for the time interval between two succeeding observations. For each scenarios, 100 simulated time series are calculated with the Gillespie algorithm [2] within COPASI [37] Version 4.11‐65. For each of the time series an estimator ${\hat{\theta }}^{\left(i\right)}$ is calculated. The table shows
the average of the estimators: $\text{av}=\frac{1}{100}\sum_{i=1}^{100}{\hat{\theta }}^{\left(i\right)}$
their ARE: $\text{ARE}=\frac{1}{100}\sum_{i=1}^{100}\frac{\left|{\hat{\theta }}^{\left(i\right)}-\theta^{\left(0\right)}\right|}{\theta^{\left(0\right)}}$

*medRE:* The median of the 100 relative errors
*w*1: the estimate of one time series shown by Wang *et al.* [8]
*w*2: the estimate of a second time series shown by Wang *et al.* [8] the rank of *w*1 and *w*2 among the 100 estimates of this paper with respect to relative error (details on how this rank is calculated can be found in Sections 3.2.2); and div stands for the number of time series for which no convergence could be achieved

**Table 3:** Statistics of the estimation results for an auto‐regulatory gene network – fully observed plus measurement noise

		*θ*_1_	*θ*_2_	*θ*_3_	*θ*_4_	*θ*_5_	*θ*_6_	*θ*_7_	*θ*_8_	*σ*	
(# Δ*t*)		0.1	0.7	0.35	0.3	0.1	0.9	0.2	0.1		div
(500 0.1)	av	0.105	0.679	0.413	0.39	0.094	0.909	0.397	0.208	0.451	2
*σ* = 0.5	ARE	10%	8%	26%	32%	10%	10%	108%	115%	11%	
*σ* estimated	medRE	9%	7%	21%	29%	9%	8%	85%	101%	10%	
(500 0.1)	av	0.145	0.886	0.947	0.937	0.098	0.969	0.883	0.499	1.603	1
*σ* = 1	ARE	45%	28%	171%	212%	10%	13%	343%	400%	60%	
*σ* estimated	medRE	43%	26%	171%	207%	8%	11%	323%	387%	60%	

This table shows the performance of the extended MSS method on an auto‐regulatory gene network model with measurement noise. Different measurement scenarios are considered as well as different amounts of DNA*_t_*. The initial values are DNA_0_ = 6, DNA.*P*_2_(0) = 4, and therefore DNA*_t_* = 10. *θ* ^(0)^ stands for the true parameter, # stands for number of measurement points and Δ*t* stands for the time interval between two succeeding observations. For both scenarios 100 simulated time series are calculated with the Gillespie algorithm [2] within COPASI [37] Version 4.11‐65. Normally distributed measurement noise is added to each of the simulated time courses with a standard deviation *σ* displayed in the table. For each of the time series, an estimator ${\hat{\theta }}^{\left(i\right)}$ is calculated. The table shows
the average of the estimators: $\text{av}=\frac{1}{100}\sum_{i=1}^{100}{\hat{\theta }}^{\left(i\right)}$
their ARE: $\text{ARE}=\frac{1}{100}\sum_{i=1}^{100}\frac{\left|\;\;{\hat{\theta }}^{\left(i\right)}-\theta^{\left(0\right)}\right|}{\theta^{\left(0\right)}}$

*medRE:* The median of the 100 relative errors div stands for the number of time series for which no convergence could be achieved

For comparison, Tables 2 and 3 include the estimate given by Wang *et al.* [8]. As [8] does not perform a simulation study, and hence only one estimate is provided, it is calculated which rank this estimate would obtain in comparison with the estimates in this paper with respect to the relative error. The comparison is described in detail in the previous section.

The optimisation has been performed as above. If the optimisation did not converge to an estimate within [0, 100]^8^, the result was counted as non‐converging.

#### 3.2.3 Observations

In both scenarios with 500 observations (rows 3 + 6) the extended MSS method performs well in estimating all parameters with a high accuracy. With only 100 observations (rows 2 + 5) the ARE for *θ*_5_ and *θ*_6_ increases. These ARE increase even more for the 50 (rows 1 + 4) observations scenario. This can be interpreted as a practical non‐identifiability. The same holds – although less strongly – for parameters *θ*_1_ and *θ*_2_ comparing rows 2 + 5 and rows 1 + 4. The increasing number of non‐converging time series with decreasing number of observation points further supports this thought. Changing the value of DNA*_t_* seems to have only a small effect on the quality of the estimation. Only in the case with 500 observations, it has a strong influence on the number of non‐converging time series. Except that the extended MSS method can cope equally well with different amounts of DNA*_t_*.

Testing the accuracy of the approximation in the fully observed case, indicates that for mRNA the mean is well approximated and the variance in most cases as well; for two others, DNA and *P*_2_, the mean fits well, but the variance is too high; whereas the fourth parameter, *P*(*t*), has a bias. It is worthy to note that while the approximation is therefore shown to be not strictly valid, most of the estimates still have an acceptably small relative error. This means that the approach for testing the approximation may be overly strict. Estimation may still work when the test fails. The reason for this is that the variance intuitively spoken acts as a weighting factor for the estimation. An estimation is possible with inaccurate variances, although with a certain loss in accuracy. The fact that an estimation is possible even with a very rough approximation of the variances, as in [4], supports that point.

The estimates provided by Wang *et al.* [8] generally fall within the range of the 100 results of the MSS method. This shows that the MSS method, in general, has comparable accuracy while being computationally less demanding. It would be interesting for further research to investigate the behaviour of the reversible jump algorithm on the time series for which the MSS method did not converge. The fact that the ARE is high for some parameters, but still most of the estimates are of a similar precision as the estimates of [8], can be explained by some estimates with extremely high relative error influencing the ARE negatively. This observation is supported by the small values for the median of the relative errors.

Table 3 shows how additive normally distributed measurement noise decreases the accuracy of the estimates with increasing standard deviation. Depending on the size of the measurement noise, parameters might become non‐identifiable (especially *θ*_7_, *θ*_8_ in the second row of Table 3).

Similar to the immigration‐death model, this model shows only few reactions between two time points of measurement, and therefore falls within the class of models that are hardly tractable without the extension. Therefore no results with the old MSS method are shown.

The results for the partially observed case (Table 4) underline that the extended MSS method can cope with partially observable scenarios. The effects of the practical non‐identifiability seem similar except that also *θ*_7_, *θ*_8_ are affected in the DNA*_t_* = 10 case.

**Table 4:** Statistics of the estimation results for an auto‐regulatory gene network – partially observed

		*θ*_1_	*θ*_2_	*θ*_3_	*θ*_4_	*θ*_5_	*θ*_6_	*θ*_7_	*θ*_8_	
(# Δ*t*)		0.1	0.7	0.35	0.3	0.1	0.9	0.2	0.1	
(50 1)	av	0.519	5.476	0.293	0.322	0.258	2.299	0.439	0.224	div 36
DNA*_t_* = 10	ARE	438%	689%	29%	25%	201%	196%	136%	137%	
	medRE	80%	138%	26%	15%	51%	50%	95%	113%	
	*w*1	0.102	0.47	0.44	0.214	0.04	0.326	0.4	0.156	rank 9
	*w*2	0.09	0.70	0.440	0.348	0.052	0.483	0.263	0.119	rank 1
(100 0.5)	av	0.111	0.753	0.351	0.299	0.257	2.337	0.194	0.097	div 4
DNA*_t_* = 10	ARE	21%	19%	18%	16%	185%	184%	17%	17%	
	medRE	13%	13%	17%	12%	28%	26%	13%	12%	
	*w*1	0.125	0.91	0.402	0.316	0.077	0.78	0.23	0.097	rank 26
	*w*2	0.188	0.64	0.413	0.25	0.072	0.64	0.43	0.196	rank 84
(500 0.1)	Av	0.101	0.696	0.349	0.305	0.094	0.905	0.202	0.101	div 0
DNA*_t_* = 10	ARE	8%	7%	13%	12%	9%	9%	14%	15%	
	medRE	7%	6%	10%	11%	8%	8%	11%	12%	
	*w*1	0.078	0.76	0.35	0.3	0.103	0.88	0.188	0.097	rank 7
(50 1)	av	0.421	2.275	0.36	0.327	0.394	3.358	0.197	0.101	div 22
DNA*_t_* = 2	ARE	340%	246%	26%	25%	336%	309%	35%	16%	
	medRE	21%	24%	18%	19%	36%	30%	31%	13%	
	*w*1	0.108	0.41	0.303	0.247	0.131	0.955	0.214	0.107	rank 7
	*w*2	0.079	0.56	0.383	0.332	0.073	0.82	0.228	0.099	rank 3
(100 0.5)	Av	0.105	0.687	0.359	0.309	0.298	2.552	0.199	0.1	div 8
DNA*_t_* = 2	ARE	23%	23%	29%	18%	220%	203%	25%	17%	
	medRE	21%	20%	26%	15%	25%	25%	20%	14%	
	*w*1	0.123	0.41	0.55	0.386	0.079	0.87	0.213	0.085	rank 48
	*w*2	0.103	0.81	0.419	0.421	0.102	1.04	0.142	0.097	rank 13
(500 0.1)	av	0.107	0.623	0.302	0.315	0.092	0.895	0.215	0.096	div 8
DNA*_t_* = 2	ARE	17%	23%	32%	25%	11%	7%	34%	16%	
	medRE	14%	19%	27%	18%	10%	5%	28%	16%	
	*w*1	0.075	0.75	0.37	0.3	0.13	0.96	0.25	0.11	rank 14

This table shows the performance of the extended MSS method on a partially observed auto‐regulatory gene network model, only mRNA, *P* and *P*_2_ observed, and compares it with results calculated by Wang *et al.* [8]. Different measurement scenarios are considered as well as different amounts of DNA*_t_*. The initial values are for DNA*_t_* = 10: DNA_0_ = 6, DNA.*P*_2_(0) = 4 as well as for DNA*_t_* = 2: DNA_0_ = 2, DNA.*P*_2_(0) = 0. *θ* ^(0)^ stands for the true parameter, # stands for number of measurement points and Δ*t* stands for the time interval between two succeeding observations. For each scenarios, 100 simulated time series are calculated with the Gillespie algorithm [2] within Copasi [37] Version 4.11‐65. For each of the time series, an estimator ${\hat{\theta }}^{\left(i\right)}$ is calculated. The table shows
the average of the estimators: $\text{av}=\frac{1}{100}\sum_{i=1}^{100}{\hat{\theta }}^{\left(i\right)}$
their ARE: $\text{ARE}=\frac{1}{100}\sum_{i=1}^{100}\frac{\left|\;{\hat{\theta }}^{\left(i\right)}-\theta^{\left(0\right)}\right|}{\theta^{\left(0\right)}}$

*medRE:* The median of the 100 relative errors
*w*1 is the estimate of one time series shown by Wang *et al.* [8]
*w*2 is the estimate of a second time series shown by Wang *et al.* [8] the rank of *w*1 and *w*2 among the 100 estimates of this paper with respect to relative error (details on how this rank is calculated can be found in the “Design of the simulation study section”) and div stands for the number of time series for which no convergence could be achieved

### 3.3 Lotka–Volterra

The third example is a Lotka–Volterra model which shows oscillatory behaviour. This model has been used as a test model for parameter estimation in [7] and consists of three reactions

$$Y^{\left(1\right)}⟶{\;}^{\theta_{1}}\;\;2Y^{\left(1\right)}$$

(6) $$Y^{\left(1\right)}+Y^{\left(2\right)}⟶{\;}^{\theta_{2}}\;\;2Y^{\left(2\right)}$$

$$Y^{\left(2\right)}⟶{\;}^{\theta_{3}}\;\;\emptyset$$

where *Y* ^(1)^ is the prey and *Y* ^(2)^ is the predator and *θ*_1_, *θ*_2_ and *θ*_3_ are parameters. The first reaction of (6) is the prey reproduction, the second the predator reproduction and the third is the predator death. In terms of ODEs this system reads as

$$\begin{array}{c} \frac{d}{dt}y^{\left(1\right)}(t)=\theta_{1}y^{\left(1\right)}(t)-\theta_{2}y^{\left(1\right)}(t)y^{\left(2\right)}(t)\\ \frac{d}{dt}y^{\left(2\right)}(t)=\theta_{2}y^{\left(1\right)}(t)y^{\left(2\right)}(t)-\theta_{3}y^{\left(2\right)}(t) \end{array}$$

The true parameter used in this paper is *θ* ^(0)^ = (0.5, 0.0025, 0.3).

#### 3.3.1 Considered scenarios

**Fig. 4:**
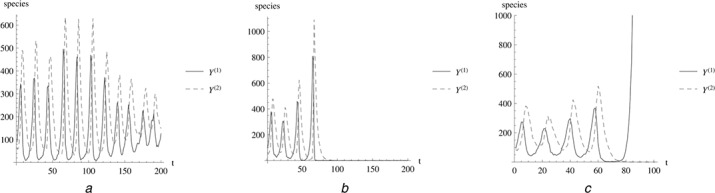
Different scenarios for Lotka–Volterra The stochastic Lotka–Volterra model can show a behaviour that is qualitatively different from the deterministic Lotka–Volterra model. Panel A shows oscillations in which the intrinsic stochasticity influences amplitude and frequency. Panel B shows a case in which the prey *Y* ^(1)^ dies out and then after that the predator *Y* ^(2)^. Panel C shows a scenario in which the predator dies out and then the prey population explodes

Five scenarios of measurement point choices are considered, three with fully observable variables and two partially observable, LVp1: recordings for *Y*_1_ and *Y*_2_(0) and LVp2: recordings for *Y*_1_ only. The fully observed scenario with total observation horizon 200 is split up in three cases depending on the specific time series behaviour: both species dying out, exploding of one species’ population or none of both, see also Fig. 4. It should be noted that on the long run asymptotically one of the species will die‐out with certainty, but the time horizon 200 is short enough to observe all three scenarios.

#### 3.3.2 Design of simulation study

For each scenario, 100 time series were created and parameter estimation was performed on each (as described for the other models). For the resulting 100 parameter estimates ${\hat{\theta }}^{\left(i\right)}$, *i* = 1, …, 100 the average $\text{av}=(1/100)\sum_{i=1}^{100}{\hat{\theta }}^{\left(i\right)}$ and the ARE $\text{ARE}=(1/100)\sum_{i=1}^{100}\left(|\;{\hat{\theta }}^{\left(i\right)}-\theta^{\left(0\right)}|/\theta^{\left(0\right)}\right)$ are displayed in Tables 5 and 6.

**Table 5:** Statistics of the estimation results for a Lotka–Volterra model – fully observed

		*θ*_1_	*θ*_2_	*θ*_3_	
(# Δ*t*)		0.5	0.0025	0.3	
(40 1)	av	0.5	0.002504	0.3	
	ARE	3%	2%	3%	
	rj	0.48	0.00255	0.308	rank 69
	bu	0.48	0.00247	0.307	rank 66
	a	0.484	0.00307	0.31	rank 101
	d	0.48	0.00254	0.307	rank 68
	old av	0.501	0.002515	0.302	
	old ARE	3%	3%	3%	
(200 1)	av	0.501	0.002499	0.3	
normal	ARE	1%	1%	1%	
	rj	0.5	0.0025	0.304	rank 10
	bu	0.5	0.00251	0.304	rank 16
	a	0.507	0.00254	0.308	rank 83
	d	0.503	0.00252	0.306	rank 52
	old av	0.5	0.002508	0.301	
	old ARE	1%	2%	2%	
(200 1)	av	0.5	0.002502	0.3	
die‐out	ARE	1%	1%	1%	
	old av	0.503	0.0025	0.299	
	old ARE	2%	2%	2%	
(200 1)	av	0.436	0.002363	0.286	
explode	ARE	13%	6%	5%	
	old av	0.214	0.001971	0.226	
	old ARE	57%	21%	25%	
(40 5)	av	0.499	0.002492	0.299	
	ARE	2%	3%	3%	
	rj	0.493	0.00262	0.314	rank 85
	bu	0.493	0.00262	0.314	rank 85
	a	0.493	0.00262	0.314	rank 85
	d	0.492	0.00263	0.315	rank 87
	old av	0.5	0.0025	0.301	
	old ARE	3%	3%	3%	

This table shows the performance of the extended MSS method on a Lotka–Volterra model and compares it with results calculated by Boys *et al.* [7]. Different measurement scenarios are considered. The initial values are (*Y* ^(1)^, *Y* ^(2)^) = (71, 79). *θ* ^(0)^ stands for the true parameter, # stands for number of measurement points and Δ*t* stands for the time interval between two succeeding observations. For each scenarios, 100 simulated time series are calculated with the Gillespie algorithm [2] within COPASI [37] Version 4.11‐65. For each of the time series, an estimator ${\hat{\theta }}^{\left(i\right)}$ is calculated. The table shows
the average of the estimators: $\text{av}=\frac{1}{100}\sum_{i=1}^{100}{\hat{\theta }}^{\left(i\right)}$
their ARE: $\text{ARE}=\frac{1}{100}\sum_{i=1}^{100}\frac{\left|\;{\hat{\theta }}^{\left(i\right)}-\theta^{\left(0\right)}\right|}{\theta^{\left(0\right)}}$
(rj) an estimate by Boys *et al.* [7] using a reversible jump method (bu) an estimate by Boys *et al.* [7] using a block updating method an estimate by Boys *et al.* [7] using an approximation of a block updating method (d) an estimate by Boys *et al.* [7] using a diffusion approximation the rank of rj, bu, a and d among the 100 estimates of this paper with respect to relative error (details on how this rank is calculated can be found in the “Design of the simulation study section”)

**Table 6:** Statistics of the estimation results for a Lotka–Volterra model – partially observed

		*θ*_1_	*θ*_2_	*θ*_3_	
(# Δ*t*)		0.5	0.0025	0.3	
(40 1)	av	0.494	0.002536	0.307	
*y*1 and *y*2(0)	ARE	6%	7%	9%	
	rj	0.469	0.00244	0.287	rank 34
	bu	0.469	0.00244	0.287	rank 34
	a	0.49	0.00236	0.273	rank 49
	d	0.473	0.00256	0.304	rank 11
	old av	0.495	0.002554	0.31	
	old ARE	6%	8%	10%	
(40 1)	av	0.491	0.002639	0.319	
only *y*1	ARE	12%	15%	17%	
	bu	0.572	0.00201	0.236	rank 39
	a	0.752	0.00151	0.176	rank 78
	d	0.432	0.00291	0.347	rank 32
	old av	0.552	0.002628	0.319	
	old ARE	27%	23%	25%	

This table shows the performance of the extended MSS method on a partially observed Lotka–Volterra model and compares it with results calculated by Boys *et al.* [7]. Different measurement scenarios are considered. The initial values are (*Y* ^(1)^, *Y* ^(2)^) = (71, 79). *θ* ^(0)^ stands for the true parameter, # stands for number of measurement points and Δ*t* stands for the time interval between two succeeding observations. For each scenarios, 100 simulated time series are calculated with the Gillespie algorithm [2] within COPASI [37] Version 4.11‐65. For each of the time series, an estimator ${\hat{\theta }}^{\left(i\right)}$ is calculated. The table shows
the average of the estimators: $\text{av}=\frac{1}{100}\sum_{i=1}^{100}{\hat{\theta }}^{\left(i\right)}$
their average relative error: $\text{ARE}=\frac{1}{100}\sum_{i=1}^{100}\frac{\left|\;{\hat{\theta }}^{\left(i\right)}-\theta^{\left(0\right)}\right|}{\theta^{\left(0\right)}}$
(rj) an estimate by Boys *et al.* [7] using a reversible jump method (bu) an estimate by Boys *et al.* [7] using a block updating method an estimate by Boys *et al.* [7] using an approximation of a block updating method (d) an estimate by Boys *et al.* [7] using a diffusion approximation the rank of rj, bu, a and d among the 100 estimates of this paper with respect to relative error (details on how this rank is calculated can be found in “Design of the simulation study sections”)

For comparison, Tables 5 and 6 include the estimates by Boys *et al.* [7]. Since in [7] only estimations from one time series are provided, we report which rank this estimate would obtain in comparison with the estimates in this paper, as described above.

The optimisation has been performed with the FindMinimum routine implemented in the software Mathematica [38] Version 8.0.4.0.

#### 3.3.3 Observations

Tables 5 and 6 show that the extended MSS method estimates all parameters in all considered scenarios including the partially observable case with a very high accuracy.

For the fully observable case with 200 observations, the study distinguishes between trajectories in which both species die‐out within the observation horizon (die‐out), trajectories in which one of the trajectories explodes (explode) and trajectories for which none of both happens (normal). One reason for the slightly higher ARE for the exploding trajectories might be that the ‘explosion’ happens before time 200, which means that the trajectories are usually shorter than the others.

Testing the approximation for the fully observed case indicates that it works very well for all ‘normal’ scenarios. For species which die‐out there is of course a huge mass of the distribution of *r* ^(*k*)^ close to zero and for species’ population that are exploding the left tail of the distribution is heavier than that of an *N*(0, 1) distribution. Nevertheless all estimations had a small relative error, again supporting the point from the previous section that the test might be overly strict, and therefore a positive test rather be used in support of the approximation, but a negative result not necessarily for a rejection.

For the partial observable case, the values for *y* ^(2)^ being not observable, the accuracy of the estimates decreases; however, still all parameters are well identifiable.

Compared with the approaches mentioned in [7], the extended MSS method does not estimate the parameters with worst accuracy.

Comparing it with the old MSS approach with constant variances, the ARE is very similar with the exception of the ‘die‐out’ scenario and the partially observed scenario, in which the extension performs better. The reason why the difference in performance is so small here is that the trajectories are highly dynamical because of the oscillations. This makes the influence of the variance – acting as a weighting factor – less strong.

## 4 Discussion and conclusion

This paper suggests an extension to the MSSs method of [4]. The extension also uses a Gaussian approximation for the transition probabilities, but in contrast to the ‘old’ version does not assume constant variances. Mean and variance are calculated by an LNA between the time points of measurements. The fact that the system is approximated only on the relatively short time intervals (with respect to the total observation horizon) allows to deal with intrinsic stochastic effects.

On the oscillatory Lotka–Volterra model, the extension performed slightly better than the old approach. The real benefit could be seen in the non‐oscillatory immigration‐death example. Here, the old versions failed in most of the scenarios to estimate the parameters correctly. The reason has already been discussed in [4] and is that only very few reactions happen between two measurements. The extension outperforms the old version considerably as it is able to estimate the parameters with small ARE for all scenarios. As the prokaryotic auto‐regulatory network falls within the scenarios where the original MSS is not expected to perform well, only the extended MSS method is tested and is shown to work fine. Depending on the choice of sample points, some parameters become non‐identifiable especially in the partially observed cases.

The suggested approach for testing the accuracy of the approximation turned out to be a candidate for a sufficient criterion. All time courses with an acceptably small relative error had as well a distribution of the *r* ^(*k*)^ which was close to a standard normal distribution. However, it does not work as a necessary criterion as there were time courses with a *r* ^(*k*)^ distribution not close to standard normal distribution having a small relative error. The reason is that the variance intuitively spoken acts as a weighting factor for the estimation, so even with a rough approximation of the variance an estimation can be possible, as shown in [4]. Further research on developing a criterion distinguishing between high relative errors because of approximation error and high relative error because of identifiability problems would be desirable.

To investigate whether the approximation leads to a loss of accuracy, the results of the method are compared with other methods. Wang *et al.* [8] uses a reversible jump technique combined with a stochastic gradient descent and Boys *et al.* [7] provides four estimates in a Bayesian framework using a reversible jump techniques and approximations. Both papers do not provide a simulation study estimating the parameters for multiple time series of the same setting. Hence, it is not possible to compare the ARE, but only to assign a rank which the provided estimate would obtain in comparison with the estimates of this paper. This comparison shows that the approximation does not lead to a significant loss in accuracy. In the case of the Lotka–Volterra oscillator even the special cases of a dying out or exploding population can be treated successfully.

Concerning the identifiability issues, techniques of experimental design would be desirable to obtain information on the identifiability of parameters as well as on confidence intervals. This is ongoing research.

Both the old and the extended MSS methods do not need stochastic simulations or the solution of a CME system. The old MSS method needs the solution of *D* ODEs (*D* number of different substances in the system) for the calculation of a transition probability. The extended MSS method requires the solution of *D* + ((*D* + 1)*D*)/2 equations, which is more, but still not prohibitive for tackling systems of the size that could be realistically treated, for example, with estimation methods for deterministic models.

For the same reasons, we expect that the MSS methods perform well from a computational performance point of view. For each evaluation of the likelihood function, one numerical integration of the system of differential equations is required. No sampling as approximation of distributions is performed. Although it will be highly model dependent how the speed of one integration compares with that of one stochastic simulation, it is definitely advantageous that the integration is only performed once per evaluation of the likelihood function, and that no trade‐off between sample numbers and accuracy needs to be considered. Furthermore, the method is comparatively easy to implement; the most complex part being a numerical integrator, for which reliable packages are readily available.
